# Lack of the association between height and cardiovascular prognosis in hypertensive men and women: analysis of national real-world database

**DOI:** 10.1038/s41598-022-22780-2

**Published:** 2022-11-08

**Authors:** Hack-Lyoung Kim, Yonggu Lee, Jun Hyeok Lee, Jeong-Hun Shin, Jinho Shin, Ki-Chul Sung

**Affiliations:** 1 Division of Cardiology, Department of Internal Medicine, Boramae Medical Center, Seoul National University College of Medicine, Seoul, South Korea; 2 Division of Cardiology, Department of Internal Medicine, Hanyang University Guri Hospital, Gyeonggi-Do, South Korea; 3 Center of Biomedical Data Science, Wonju College of Medicine, Yonsei University, Wonju, South Korea; 4 Division of Cardiology, Department of Internal Medicine, Hanyang University College of Medicine, 222 Wangsimni-Ro Sungdong-Gu, Seoul, 04763 South Korea; 5 Division of Cardiology, Department of Internal Medicine, Kangbuk Samsung Hospital, Sungkyunkwan University School of Medicine, 29 Saemunan-Ro, Jongno-Gu, Seoul, 03181 South Korea

**Keywords:** Anatomy, Cardiology

## Abstract

Data on the association between height and cardiovascular risk are still conflicting. Moreover, no reports are showing this issue in hypertensive patients. This study was performed to investigate whether height affects cardiovascular prognosis in hypertensive patients using nation-wide real-world data. Using the Korean National Health Insurance Service database, we analyzed 461,492 Korean hypertensive patients without any prior history of cardiovascular disease between January 2002 and December 2017. The incidence of a composite of cardiovascular death, myocardial infarction, and stroke was assessed according to height quintiles. In univariable comparisons, the taller the patients, the younger the age and the higher the proportion of men. In multivariable cox regression analyses, height was not associated with the occurrence of cardiovascular events. Although the risk of clinical events increased in some height quintiles compared to the first height quintile, there was no tendency to increase the risk according to the increase in the height quintile. These results were similar even when men and women were analyzed separately. In the same quintile group of height, there were no significant differences in clinical outcomes between sexes. In Korean hypertensive patients, there was no association between height and the occurrence of cardiovascular events. This result did not differ by sex. The clinical use of height for CVD prediction seems to be still tricky in hypertensive patients.

## Introduction

An inverse association between height and cardiovascular risk has been reported in many epidemiological studies^1–6^ and meta-analyses^7, 8^. As a potential mechanism, it has been suggested that short stature is associated with increased blood pressure (BP)^9, 10^, arterial stiffness^11–13^, and unfavorable lipid and glucose metabolism^3^. More specifically, in terms of arterial stiffness, it was reported that the aortic pressure augmentation increases in short stature, which leads to a decrease in pulse pressure amplification in peripheral arteries^13^. However, some other studies have shown no association between height and cardiovascular prognosis^14–17^, or even a positive association^18, 19^.

Hypertension has a very high prevalence and is the number one cause of death as a single disease^20^. In hypertensive patients, finding factors that predict CVD in addition to BP control and providing customized treatment is important^21^. One of the primary rationales behind the hypothesized association between height and cardiovascular risk is the pulse pressure amplification of the aorta, which also largely depends on its stiffness^22^. In patients with hypertension, aorta stiffness is supposedly more significant than in the general population; therefore, height may have a different impact on the pulse pressure amplification in hypertensive patients than in normotensive patients. However, to date, no studies have reported height’s influence on hypertensives’ cardiovascular risk. Since information about height is easily obtainable, research on this issue will be of great significance to clinicians treating patients with hypertension.

This study aimed to investigate whether height affects cardiovascular prognosis in hypertensive patients using nationwide cohort data. We also analyzed sex differences on the same issue.

## Methods

### Data sources

This study used the Korean National Health Insurance Service (NHIS) national health screening database. In South Korea, the NHIS operates the National Health Insurance (NHI), which covers 97% of Korean population. The NHIS also conducts national health screening every two years for healthy Koreans over the age of 40. The NHIS national health screening database provides data about demographics, socioeconomics, medical treatments, medical procedures, diagnoses, prescriptions, health questionnaires, health screening laboratory tests and date of death and cause of death^23^. The study was approved by the Institutional Review Board (IRB) of Kangbuk Samsung Hospital (Seoul, South Korea) (# KBSMC 2022-01-056). Obtaining informed consent from study subjects was waived by the IRB. All study procedures and processes were conducted in accordance with the Declaration of Helsinki, revised in 2013.

### Enrollment of study patients

**Figure 1:**
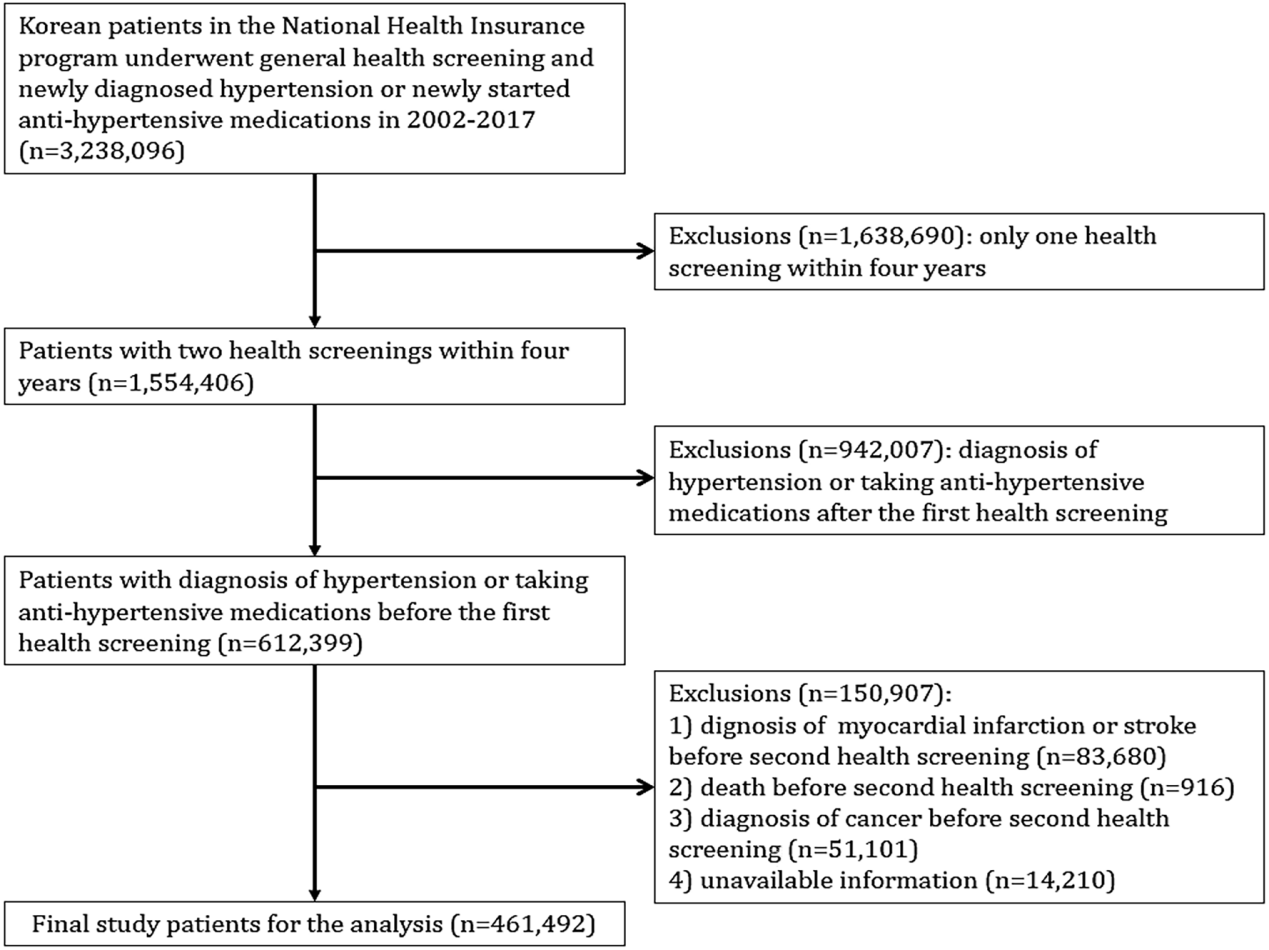
Flow chart for the enrollment of study patients.

Between January 2002 and December 2017, 3,238,096 patients were diagnosed with hypertension (the 10th International Classification of Disease [ICD]-10 code: I10-I15) or received anti-hypertensive medications in the NHIS national health screening database. The baseline clinical information was obtained from two sequential health screening data of NHIS within four years between 2002 and 2011. Clinical outcome data were collected from the time point after the second health screening. Therefore, 1,638,690 with only one health screening within four years, and 942,007 with a diagnosis of hypertension or taking anti-hypertensive medications after the first health screening were sequentially excluded. Among the remaining 612,399, 150,907 were further excluded due to (1) diagnosis of myocardial infarction, stroke or heart failure before second health screening (*n* = 83,680), (2) death before the second health screening (*n* = 916), (3) diagnosis of cancer before the second health screening (*n* = 52,101), and (4) unavailable information for study analysis (*n* = 14,210). After these exclusions, 461,492 patients were finally analyzed. The flow chart for study enrollment is shown in Fig. 1.

### Clinical data collection

Height was measured from the floor in a standing position with shoes off. Body mass index was obtained by dividing weight by the square of height (kg/m^2^). BP was measured using an oscillometric device on the right upper arm. Information on lifestyle habits, such as smoking, drinking alcohol, and exercise, was obtained through a health screening questionnaire. Because a health insurance premium is set in proportion to income, we classified household income levels based on the health insurance premium. Information about diabetes mellitus was obtained using the diagnostic codes (ICD-10 codes: E10-E14). After an overnight fast, venous blood was taken from the antecubital vein, and cholesterol and glucose serum levels were measured. Information on the use of aspirin and statin was also obtained.

### Cardiovascular events

The primary study endpoint was a major cardiovascular event (MACE), defined as a composite of cardiovascular death, myocardial infarction, stroke, and heart failure. Each clinical event served as the secondary endpoint. The clinical events were identified using the ICD-10 codes registered in the Korean National Health Insurance Service System (KNHISS) for reimbursement when patients were discharged from a hospital (I21-23 for myocardial infarction, I60-69 for stroke [including both hemorrhagic and ischemic stroke] and I50 for heart failure). In addition, the cause and date of death were identified using the death certificate registry database from the National Statistical Office of Korea.

### Statistical analysis

Continuous variables are expressed as mean (SD) and categorical variables as n (%). Height was divided equally into five groups at quintile values. Continuous and categorical variables were compared among groups using the analysis of variance and the Chi-square test, respectively. The clinical event incidence was calculated using the total number of outcomes during the follow-up period divided by 100,000 person-years. Multivariable analysis was performed using the Cox proportional hazard model to evaluate the relationship between height and cardiovascular events. The hazard ratio (HR) and 95% confidence interval (CI) for the cardiovascular events were calculated and adjusted for age, body mass index, systolic and diastolic BPs, diabetes mellitus, smoking, alcohol consumption, physical activity, income level, fasting glucose, total cholesterol, and use of medications (aspirin, statin, and antihypertensive medications) in each height group against the first quintile group. Sensitivity analyses for the multivariable Cox proportional hazard models were conducted with the height groups divided by sex-specific quintile height values. Statistical analyses were performed using SAS Software (version 9.4, SAS Institute, Cary, NC, USA) and R Statistical Software (version 3.5.2, R Foundation for Statistical Computing, Vienna, Austria).

## Results

### Baseline characteristics of the study patients

The baseline characteristics of the study patients according to height are demonstrated in Table 1. Patients were younger and more likely to be males in the higher quintiles than in the lower quintiles. Systolic BP was highest in the first quintile group, and diastolic BP was highest in the fifth quintile group. Therefore, pulse pressure was highest in the first quintile group and lowest in the fifth quintile group. The patients were more likely to drink alcohol and smoke and have higher physical activities in the higher quintiles than in the lower quintiles. The patients in the higher quintiles had higher blood glucose levels but lower cholesterol levels than those in the lower quintiles of height.

**Table 1:** Baseline characteristics of study patients.

Characteristic	Height quintile					*P* value
	1st (< 151 cm)	2nd (151–155.9 cm)	3rd (156–161.9 cm)	4th (162–167.9 cm)	5th (≥ 168 cm)	
n (%)	81,084 (17.6)	89,291 (19.4)	102,661 (22.2)	89,268 (19.3)	99,188 (21.5)	< 0.001
Age, median (IQR), years	67 (60–72)	60 (54–68)	58 (50–66)	58 (50–66)	52 (44–62)	< 0.001
Female sex, n (%)	80,189 (98.9)	84,349 (94.5)	72,868 (71.0)	19,402 (21.7)	2,066 (2.1)	< 0.001
Male sex, n (%)	895 (1.1)	4,942 (5.5)	29,793 (29.0)	69,866 (78.3)	97,122 (97.9)	< 0.001
Body mass index, mean (SD), kg/m^2^	24.9 (3.5)	24.9 (3.3)	24.6 (3.2)	24.6 (3.1)	25.1 (3.2)	< 0.001
< 18.5, n (%)	1,886 (2.3)	1,352 (1.5)	1,722 (1.7)	1,635 (1.8)	1,186 (1.2)	< 0.001
18.5–22.9, n (%)	21,031 (25.9)	23,885 (26.8)	29,541 (28.8)	24,179 (27.1)	21,705 (21.9)	
23.0–24.9, n (%)	20,079 (24.8)	21,181 (23.7)	26,546 (25.8)	23,766 (26.6)	26,092 (26.3)	
≥ 25.0, n (%)	38,088 (47.0)	42,873 (48.0)	44,852 (43.7)	39,688 (44.5)	50,205 (50.6)	
Systolic BP, mean (SD), mmHg	134.3 (12.8)	132.2 (12.9)	131.7 (13.2)	132.9 (12.8)	132.9 (12.2)	< 0.001
Diastolic BP, mean (SD), mmHg	80.9 (7.6)	80.6 (7.8)	80.8 (8.1)	81.9 (8.1)	83.0 (8.1)	< 0.001
Smoking						< 0.001
Never, n (%)	78,163 (96.4)	83,949 (94.0)	83,054 (80.9)	45,766 (51.3)	34,535 (34.8)	
Past, n (%)	926 (1.1)	2,151 (2.4)	9,891 (9.6)	22,952 (25.7)	31,796 (32.1)	
Current, n (%)	1,995 (2.5)	3,191 (3.6)	9,716 (9.5)	20,550 (23.0)	32,857 (33.1)	
Alcohol consumption, times/week						< 0.001
0, n (%)	73,297 (90.4)	75,948 (85.1)	74,725 (72.8)	44,352 (49.7)	35,197 (35.5)	< 0.001
< 1, n (%)	4,374 (5.4)	6,911 (7.7)	11,410 (11.1)	14,100 (15.8)	20,313 (20.5)	
1–2, n (%)	2,319 (2.9)	4,456 (5.0)	10,565 (10.3)	19,418 (21.7)	29,473 (29.7)	
3–4, n (%)	445 (0.5)	867 (1.0)	2,845 (2.8)	5,963 (6.7)	8,536 (8.6)	
≥ 5	649 (0.8)	1109 (1.2)	3116 (3.0)	5435 (6.1)	5,669 (5.7)	
Physical activity, times/week						< 0.001
0, n (%)	55,766 (68.8)	53,546 (60.0)	55,831 (54.4)	42,520 (47.7)	40,260 (40.6)	
1–2, n (%)	8,603 (10.6)	11,059 (12.4)	14,110 (13.7)	14,159 (15.8)	19,987 (20.1)	
3–4, n (%)	6,203 (7.6)	9,289 (10.4)	11,918 (11.6)	11,831 (13.2)	15,261 (15.4)	
5–6, n (%)	4,264 (5.3)	6,520 (7.3)	8,889 (8.7)	8,647 (9.7)	10,322 (10.4)	
7, n (%)	6,248 (7.7)	8,877 (9.9)	11,913 (11.6)	12,111 (13.6)	13,358 (13.5)	
Household income, quartile						< 0.001
First (highest), n (%)	28,029 (34.6)	29,970 (33.6)	33,889 (33.0)	30,347 (34.0)	34,559 (34.8)	
Second, n (%)	19,457 (24.0)	21,674 (24.3)	25,114 (24.5)	22,195 (24.9)	26,414 (26.6)	
Third, n (%)	15,087 (18.6)	16,619 (18.6)	19,719 (19.2)	17,511 (19.6)	20,170 (20.4)	
Fourth (lowest), n (%)	18,511 (22.8)	21,028 (23.5)	23,939 (23.3)	19,215 (21.5)	18,045 (18.2)	
Diabetes mellitus, n (%)	9,181 (11.3)	9,758 (10.9)	10,996 (10.7)	10,192 (11.4)	10,573 (10.6)	< 0.001
Fasting glucose, mean (SD), mg/dL	103.7 (28.2)	103.5 (27.7)	104.1 (28.9)	107.0 (31.4)	108.2 (33.7)	< 0.001
< 100.0	45,729 (56.4)	51,040 (57.2)	58,106 (56.6)	45,340 (50.8)	49,055 (49.5)	< 0.001
100.0–125.9	25,850 (31.9)	27,981 (31.3)	32,108 (31.3)	30,713 (34.4)	34,604 (34.9)	
≥ 126.0	9,505 (11.7)	10,270 (11.5)	12,447 (12.1)	13,215 (14.8)	15,529 (15.6)	
Total cholesterol, mean (SD), mg/dL	204.0 (41.3)	201.8 (43.6)	197.4 (41.1)	191.9 (39.8)	191.8 (38.8)	< 0.001
< 200.0	39,098 (48.2)	45,299 (50.7)	56,588 (55.1)	54,354 (60.9)	60,411 (60.9)	< 0.001
200.0 ~ 239.9	27,862 (34.4)	29,718 (33.3)	32,532 (31.7)	25,764 (28.9)	28,902 (29.1)	
≥ 240.0	14,124 (17.4)	14,274 (16.0)	13,541 (13.2)	9,150 (10.2)	9,875 (10.0)	
Aspirin, n (%)	20,724 (25.6)	21,828 (24.5)	24,657 (24.0)	23,053 (25.8)	23,405 (23.6)	< 0.001
Statin, n (%)	10,808 (13.3)	13,296 (14.9)	13,597 (13.2)	10,746 (12.0)	11,636 (11.7)	< 0.001
Antihypertensive medication, n (%)	27,066 (33.4)	29,904 (33.5)	32,607 (31.8)	28,502 (31.9)	29,389 (29.6)	< 0.001

*IQR* interquartile range, *SD* standard deviation, *BP* blood pressure.

### Clinical outcome according to height

**Figure 2:**
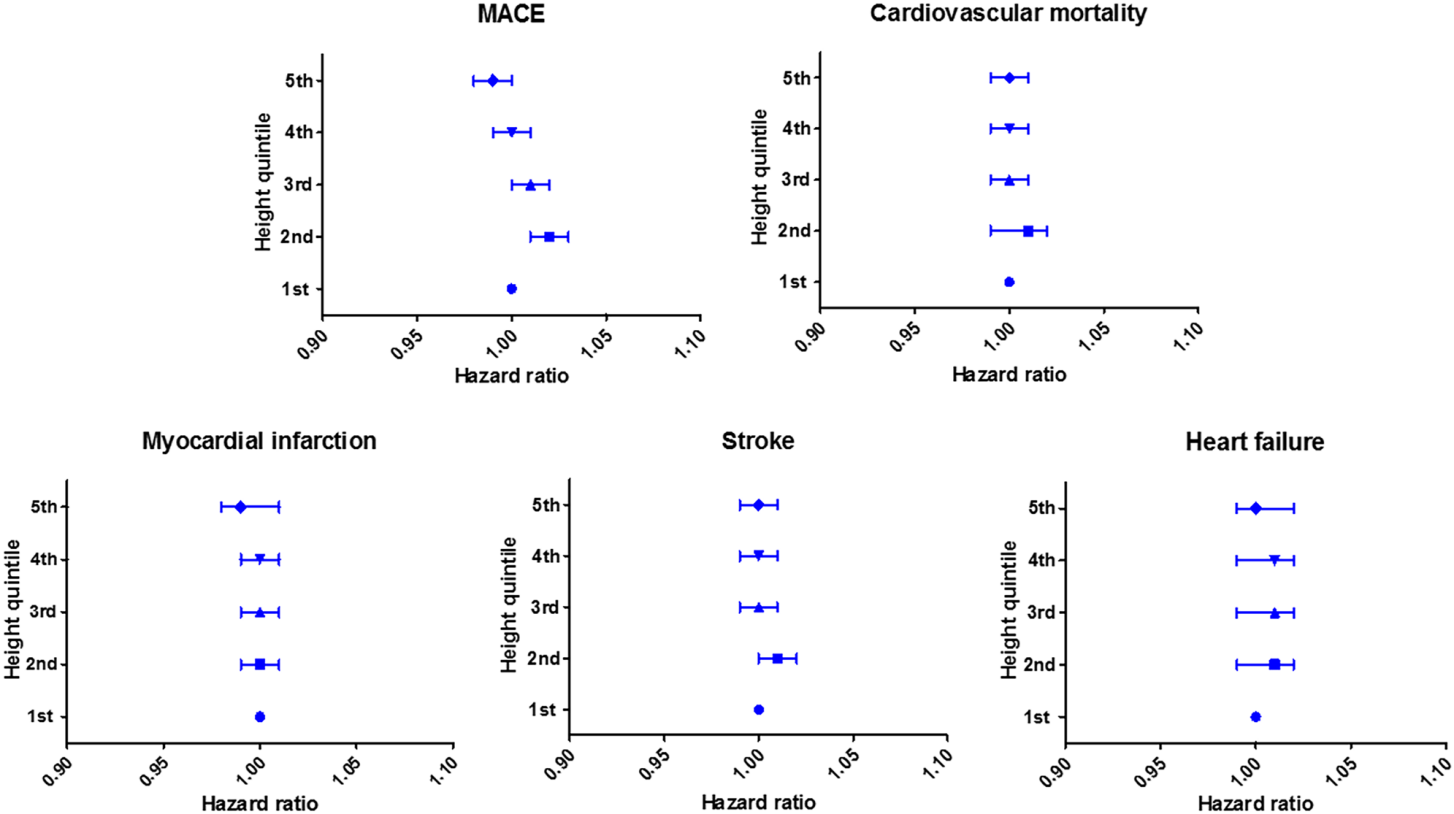
The risk of MACE and each cardiovascular event according to height quintile. *MACE* major adverse cardiovascular event.

**Figure 3:**
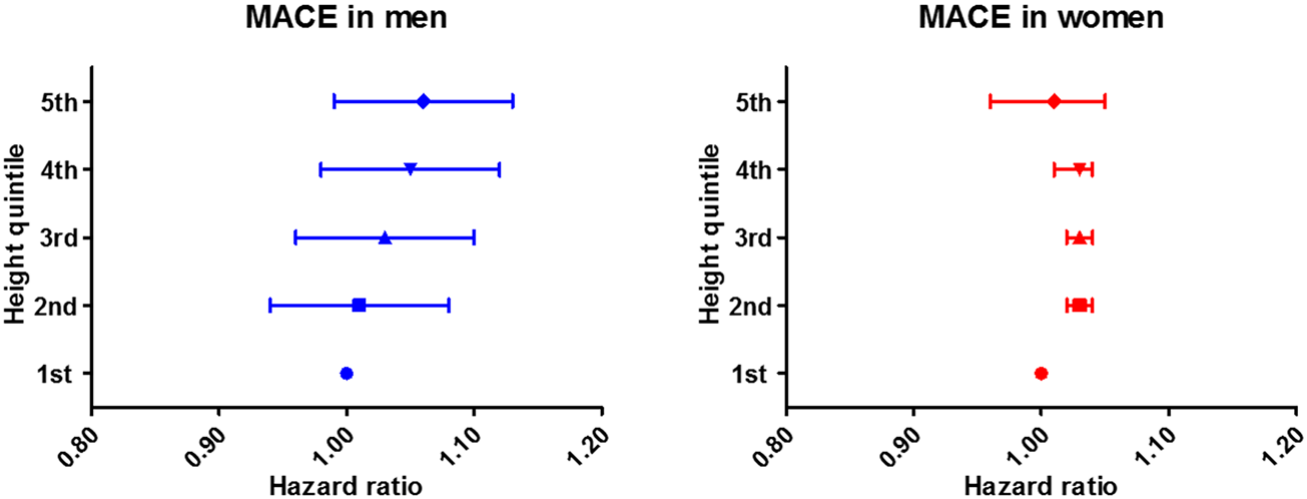
The risk of MACE according to height quintile in men and women. *MACE* major adverse cardiovascular event.

Adjusted risks of cardiovascular events according to the height groups are shown in Table 2. Compared to the first quintile group (lowest height), the second (HR, 1.02; 95% CI, 1.01–1.03) and the third (HR, 1.01; 95% CI, 1.00–1.02) quintile group had higher risks of MACE, whereas the fourth (HR, 1.00; 95% CI, 0.99–1.01) and the fifth (HR, 0.99; 95% CI, 0.98–1.00) quintile groups did not. No associations were found between height and the risks of other cardiovascular events, including cardiovascular mortality, myocardial infarction, stroke, and heart failure, except for increased risk of stroke in patients with the second quintile group than in those with the first quintile group (HR, 1.01; 95% CI, 1.00–1.02). The risk of MACE and each clinical event according to height quintiles are demonstrated in Fig. 2. In the sensitivity analyses with the groups classified using the sex-specific quintile values, the risks of MACE and individual cardiovascular events were not associated with the height groups in men (Supplementary Table S1, Fig. 3). In women, the second, third and fourth quintile groups were more significantly associated with higher risks of MACE than the first quintile group, but there was no trend in the risks of MACE among the height groups (Supplementary Table S2, Fig. 3). The risks of cardiovascular events according to height in women compared to men are shown in Table 3. In the first and fifth quintile groups, there were no significant differences in any clinical outcomes between sexes. In the second, third, and fourth quintile groups, women tended to have higher risks of MACE, cardiovascular mortality and, stroke in groups aged ≥ 50 years than men in the same height quintile and age groups.

**Table 2:** Clinical outcome according to height.

Clinical outcome	Height quintile				
	1st (lowest)	2nd	3rd	4th	5th (highest)
**MACE**					
Events	15,572	11,455	12,712	12,671	10,947
Person-years	691,632	781,759	899,592	796,954	907,980
Incidence (events/100,000 person-years)	2251	1465	1413	1590	1206
Adjusted HR (95% CI)	Ref	1.02 (1.01–1.03)	1.01 (1.00–1.02)	1.00 (0.99–1.01)	0.99 (0.98–1.00)
**Cardiovascular mortality**					
Events	3110	1717	1990	2150	1590
Person-years	738,065	821,823	937,053	828,760	937,638
Incidence (events/100,000 person-years)	421	209	212	259	170
Adjusted HR (95% CI)	Ref	1.01 (0.99–1.02)	1.00 (0.99–1.01)	1.00 (0.99–1.01)	1.00 (0.99–1.01)
**Myocardial infarction**					
Events	1575	1215	1628	1879	1952
Person-years	773,434	841,542	964,914	861,613	961,218
Incidence (events/100,000 person-years)	204	144	169	218	203
Adjusted HR (95% CI)	Ref	1.00 (0.99–1.01)	1.00 (0.99–1.01)	1.00 (0.99–1.01)	0.99 (0.98–1.01)
**Stroke**					
Events	8839	6806	7523	7427	6120
Person-years	727,814	806,699	928,110	826,380	934,721
Incidence (events/100,000 person-years)	1214	844	811	899	655
Adjusted HR (95% CI)	Ref	1.01 (1.00–1.02)	1.00 (0.99–1.01)	1.00 (0.99–1.01)	1.00 (0.99–1.01)
**Heart failure**					
Events	4183	2891	2893	2577	2245
Person-years	759,110	832,727	958,415	858,226	960,157
Incidence (events/100,000 person-years)	551	347	302	300	234
Adjusted HR (95% CI)	Ref	1.01 (0.99–1.02)	1.01 (0.99–1.02)	1.01 (0.99–1.02)	1.00 (0.99–1.02)

*MACE* major adverse cardiovascular event, *HR* hazard ratio, *CI* confidence interval, *Ref.* reference.

**Table 3:** Women’s risk for cardiovascular events according to height compared to men.

Height quintile	Clinical outcome				
	MACE	CV mortality	MI	Stroke	HF
**1st (lowest)**					
Total	1.05 (0.98–1.13)	1.04 (0.97–1.12)	1.02 (0.95–1.09)	1.04 (0.97–1.11)	0.99 (0.92–1.06)
Age ≤ 50 years	0.98 (0.73–1.31)	1.00 (0.75–1.33)	1.01 (0.75–1.35)	0.98 (0.74–1.31)	0.99 (0.74–1.33)
Age > 50 years	1.06 (0.99–1.14)	1.04 (0.97–1.12)	1.02 (0.95–1.09)	1.04 (0.97–1.12)	0.99 (0.92–1.06)
**2nd**					
Total	1.08 (1.04–1.12)	1.03 (1.00–1.07)	1.02 (0.98–1.05)	1.05 (1.01–1.08)	1.01 (0.97–1.04)
Age ≤ 50 years	1.08 (0.96–1.21)	1.00 (1.00–1.00)	1.03 (0.91–1.16)	1.04 (0.92–1.17)	1.02 (0.90–1.15)
Age > 50 years	1.07 (1.04–1.11)	1.03 (1.00–1.07)	1.02 (0.98–1.05)	1.04 (1.01–1.08)	1.01 (0.97–1.04)
**3rd**					
Total	1.08 (1.06–1.10)	1.03 (1.01–1.05)	1.02 (1.00–1.04)	1.05 (1.03–1.07)	1.01 (0.99–1.03)
Age ≤ 50 years	1.03 (0.99–1.08)	1.00 (0.96–1.05)	1.01 (0.97–1.06)	1.02 (0.97–1.06)	1.00 (0.96–1.05)
Age > 50 years	1.09 (1.06–1.11)	1.03 (1.01–1.05)	1.02 (1.00–1.04)	1.05 (1.03–1.07)	1.01 (0.99–1.03)
**4th**					
Total	1.07 (1.05–1.09)	1.01 (0.99–1.03)	1.02 (1.00–1.04)	1.03 (1.01–1.05)	1.01 (0.99–1.03)
Age ≤ 50 years	1.03 (1.00–1.07)	1.00 (0.97–1.04)	1.01 (0.98–1.05)	1.01 (0.98–1.05)	1.01 (0.97–1.04)
Age > 50 years	1.08 (1.05–1.11)	1.02 (0.99–1.04)	1.02 (0.99–1.05)	1.04 (1.02–1.07)	1.01 (0.98–1.03)
**5th (highest)**					
Total	1.04 (0.99–1.09)	1.01 (0.96–1.05)	1.01 (0.97–1.06)	1.01 (0.97–1.06)	1.01 (0.97–1.06)
Age ≤ 50 years	1.02 (0.96–1.08)	1.00 (0.95–1.06)	1.01 (0.95–1.07)	1.01 (0.95–1.07)	1.00 (0.95–1.06)
Age > 50 years	1.06 (0.98–1.15)	1.01 (0.94–1.09)	1.02 (0.94–1.10)	1.02 (0.95–1.11)	1.02 (0.94–1.10)

*MACE* major adverse cardiovascular event, *CV* cardiovascular, *MI* myocardial infarction, *HF* heart failure.

## Discussion

In this nationwide real-world study of Korean hypertensive patients, height was not associated with the occurrence of cardiovascular events. This lack of association did not vary by sex. To the best of our knowledge, this is the first study showing the association between height and CVD risk in hypertensive patients.

Numerous studies have shown a negative association between height and the risk of developing cardiovascular events^1–6^. Rich-Edwards et al. investigated 121,700 female nurses in the USA and showed that, compared with the shortest women with height ≤ 155 cm, the relative risks of coronary heart disease decreased by 18%, 26%, 21%, and 27% for 157–160 cm, 163 cm, 165–168 cm and ≥ 170 cm tall women, respectively^5^. In a Japanese study that followed 15,564 people for 16 years, short stature was associated with an increased risk of stroke in both men and women^1^. In another study, short stature was an independent predictor of MACE among 1490 patients with ST-elevation myocardial infarction undergoing percutaneous coronary intervention^2^. The association between genetically determined height and the occurrence of coronary artery disease has also been reported. Nelson et al. obtained data on 180 height-associated genetic variants and showed a graded relationship between the presence of an increased number of height-raising variants and a reduced risk of coronary artery disease^3^. In a Danish population-based cohort study that followed 12,859 men for 36 years, shorter men had a 33% higher risk of ischemic heart disease than tall men^6^. A recent Korean study of 16,528,128 subjects who underwent health check-ups showed an inverse relationship between height and the occurrence of myocardial infarction, heart failure, stroke, and all-cause mortality during a 9-year follow-up period, regardless of age and sex^4^. That study is similar to ours in that it targeted Koreans with the same data source, but there is a significant difference in the study population. Our study included only patients with hypertension, who are older and supposedly have higher arterial stiffness than the general population in the study performed by Park et al.^4^ The range of age and height were also narrower in our study than in their study. This higher arterial stiffness and narrower height range may have caused a different impact of height on cardiovascular pathophysiology and eventually resulted in a different risk of CVD^22, 24^. Additionally, unlike our study, some of the important clinical covariates, such as cardiovascular medications, were not adjusted during multivariable analysis in the study performed by Park et al.^4^ Another important difference was that the effect of height itself on cardiovascular events, regardless of sex, was shown only in our study. Interest in sex differences in the CVD field continues to grow. It has been identified that CVD has sex-related aspects, and understanding these differences and tailoring treatment can help improve the patient’s prognosis^25^. We additionally provided data on the sex-specific risk for cardiovascular outcomes in patients with the same height category between men and women. In Korean adults, both men and women begin to decrease in height after their 40 s, but the decrease is greater in women after their 50 s (http://www.motie.go.kr/www/main.do). Considering this, we compared the differences between men and women before and after the age of 50, but there was no significant sex-related association between height and CVD occurrence.

In addition to genetic factors^3^, poor nutritional status, unfavorable lipid and glucose metabolism^3, 10, 26^, poor lung function^26^, high BP^9, 10^, increased pulsatile load to the left ventricle^3, 10–12^, and small arterial diameter leading to more occlusive events^27^ in short stature are suggested as possible mechanisms for the association between short stature and high cardiovascular risk.

However, some studies have shown a lack of association between height and CVD^14–17^. In a study of 10,427 people in Scandinavia, although the association between short height and cardiovascular risk was observed in univariate analysis, the significance of the association disappeared after adjustment for confounders^14^. In a 35-year follow-up of 4604 men and women in the Framingham Hurt study cohort, after adjusting for age and other risk factors, short stature increased cardiovascular risk in neither both men and women^15^. Similarly, Liao et al. showed a lack of association between height and cardiovascular disease risk in a multivariable analysis of a 13-year follow-up of 13,031 subjects^16^. Song et al. investigated 344,519 Korean women and showed that short stature was associated with all-cause mortality but not with mortality from ischemic heart disease and stroke^17^. Although we are limited to hypertensive individuals, our results are in line with these findings. Compared to the aforementioned Korean study^17^, our study has strengths because we excluded patients with prior CVD and controlled confounding effects of cardiovascular medications.

Research results on the relationship between height and cardiovascular risk are not consistent, and various possible mechanisms exist. Most of the existing studies showing the association between short stature and high cardiovascular risk are epidemiological ones with long-term follow-up. The average heights increased in both men and women as living standards and nutritional status improved over time^28, 29^. With improvements in welfare and advance in medical technology, life expectancy also improves over time. Given that CVD is a leading cause of death in the elderly, it is naturally hypothesized that older people with shorter stature may have a poorer cardiovascular prognosis than younger people with taller stature. Also, height may not only indicate a physical difference influencing vascular physiology but also imply socioeconomic status and childhood undernourishment or illness in developing countries^30^. Therefore, if a secular trend of height is not properly adjusted, it would be difficult to conclude a causal relationship between height and cardiovascular risk^10, 29^. Regardless of how thorough adjustments are exerted through multivariable statistical modeling, it may be impossible to eliminate the impacts caused by the recent rise in stature^29^. Only studies with long-term follow-ups of people of the same age at the same time can give a clear answer to the association between height and the incidence of CVD.

Previous studies have reported that the risk of left ventricular hypertrophy and atrial fibrillation, closely related to cardiovascular prognosis, increase with height^31, 32^. Moreover, some researchers suggested that short stature may actually increases the survival rate because of reduced telomere shortening, lower DNA damage, and higher efficacy of myocardial contractility^33^. Furthermore, there is a possibility of error in the estimation of the traveling length of pulse wave using height in studies on the association between height and pulse wave velocity, one of a marker of cardiovascular prognosis^11, 34^. Like our study, there have been several studies of a large number of Koreans, but the results were inconsistent^4, 13, 17, 28^. For these reasons, it seems that height is not yet reflected in guidelines as a predictor for CVD. We showed that height in hypertensive patients was not associated with CVD risk. Although height is a simple, measurable variable, it would be difficult to apply height to predict cardiovascular prognosis in hypertensive patients.

### Study limitations

There are several potential limitations of this study. First, as mentioned above, although the aging effect was corrected through multivariable analysis, it would have been difficult to completely exclude the bias caused by the secular trend of height increase. To overcome this bias, long-term follow-up studies with contemporaries of the same age are needed. Second, the problem of diagnostic accuracy inevitably exists due to the acquisition of information on clinical events based on diagnostic codes. Third, a decrease in height with age (especially in women) was not considered in this study. Fourth, pule pressure information was not available in our study. Pulse pressure is one of the arterial stiffness indicators, so pulse pressure data analysis would have provided additional information. Fifth, BP measurement methods were not uniform in our study. Although the Korean government suggested and educated the standard methods for BP measurement, it could not be confirmed how BP was measured for individual patients because our data was from the NHIS database. Lastly, as our study patients were restricted to Korean hypertensive patients without CVD, it is difficult to apply our results to other populations.

## Conclusions

The results of this study suggest that there may be no association between height and the occurrence of cardiovascular events in Korean hypertensive patients. The clinical use of height for CVD prediction seems to be still tricky in hypertensive patients.

## Supplementary Information

Supplementary Information.

## Data Availability

All data generated or analyzed during this study are included in this article.

## Abbreviations

BP: Blood pressure
CI: Confidence interval
CVD: Cardiovascular disease
HR: Heart rate
ICD: International classification of disease
IRB: Institutional review board
KNHISS: Korean national health insurance service system
MACE: Major cardiovascular event
NHI: National health insurance
NHIS: National health insurance service
