# Supplementary material for: Lack of the association between height and cardiovascular prognosis in hypertensive men and women: analysis of national real-world database
Source: Sci Rep. 2022 Nov 8;12:18953. doi: 10.1038/s41598-022-22780-2 (PMC9643395; doi:10.1038/s41598-022-22780-2)
Supplement: Supplementary file 1 — Supplementary Information. [file 41598_2022_22780_MOESM1_ESM.docx]

**Supplementary Table S1. Clinical outcome according to height in men**

| **Clinical outcome** | **Height quintile** | | | | |
| --- | --- | --- | --- | --- | --- |
|  | **1st**  **(<162 cm)** | **2nd**  **(162-165.9 cm)** | **3rd**  **(166-168.9 cm)** | **4th**  **(169-171.9 cm)** | **5th**  **(≥172 cm)** |
| MACE |  |  |  |  |  |
| Events | 257 | 1,286 | 6,216 | 11,462 | 10,859 |
| Person-years | 7,520 | 42,024 | 261,887 | 628,643 | 890,501 |
| Incidence (events/100000 person-years) | 3,418 | 3,060 | 2,374 | 1,823 | 1,219 |
| Adjusted HR (95% CI) | Ref. | 1.01 (0.94-1.08) | 1.03 (0.96-1.10) | 1.05 (0.98-1.12) | 1.06 (0.99-1.13) |
| Cardiovascular mortality |  |  |  |  |  |
| Events | 90 | 331 | 1,334 | 2,042 | 1,582 |
| Person-years | 7,439 | 43,586 | 272,758 | 655,663 | 919,810 |
| Incidence (events/100000 person-years) | 1,210 | 759 | 489 | 311 | 172 |
| Adjusted HR (95% CI) | Ref. | 1.03 (0.96-1.10) | 1.04 (0.97-1.11) | 1.05 (0.98-1.12) | 1.14 (0.98-1.12) |
| Myocardial infarction |  |  |  |  |  |
| Events | 37 | 180 | 926 | 1,751 | 1,941 |
| Person-years | 8,805 | 48,882 | 293,887 | 687,301 | 943,342 |
| Incidence (events/100000 person-years) | 420 | 368 | 315 | 255 | 206 |
| Adjusted HR (95% CI) | Ref. | 1.00 (0.94-1.01) | 1.01 (0.94-1.08) | 1.01 (0.95-1.08) | 1.01 (0.95-1.08) |
| Stroke |  |  |  |  |  |
| Events | 143 | 742 | 3,622 | 6,695 | 6,066 |
| Person-years | 8,171 | 45,132 | 276,499 | 655,632 | 917,077 |
| Incidence (events/100000 person-years) | 1,750 | 1,644 | 1,310 | 1,021 | 661 |
| Adjusted HR (95% CI) | Ref. | 1.00 (0.93-1.07) | 1.01 (0.95-1.08) | 1.02 (0.96-1.09) | 1.03 (0.96-1.10) |
| Heart failure |  |  |  |  |  |
| Events | 47 | 259 | 1,185 | 2,266 | 2,227 |
| Person-years | 8,778 | 483,87 | 292,551 | 684,816 | 942,286 |
| Incidence (events/100000 person-years) | 535 | 535 | 405 | 331 | 236 |
| Adjusted HR (95% CI) | Ref. | 1.00 (0.93-1.07) | 1.00 (0.94-1.07) | 1.00 (0.94-1.07) | 1.00 (0.94-1.07) |

MACE, major adverse cardiovascular event; HR, hazard ratio, CI, confidence interval; Ref., reference.

**Supplementary Table S2. Clinical outcome according to height in women**

| **Clinical outcome** | **Height quintile** | | | | |
| --- | --- | --- | --- | --- | --- |
|  | **1st**  **(<148 cm)** | **2nd**  **(148-151.9 cm)** | **3rd**  **(152-154.9 cm)** | **4th**  **(155-157.9 cm)** | **5th**  **(≥158 cm)** |
| MACE |  |  |  |  |  |
| Events | 15,315 | 10,169 | 6,496 | 1,209 | 88 |
| Person-years | 684,112 | 739,735 | 637,705 | 168,311 | 17,479 |
| Incidence (events/100000 person-years) | 2,239 | 1,375 | 1,019 | 718 | 503 |
| Adjusted HR (95% CI) | Ref. | 1.03 (1.02-1.04) | 1.03 (1.02-1.04) | 1.03 (1.01-1.04) | 1.01 (0.96-1.05) |
| Cardiovascular mortality |  |  |  |  |  |
| Events | 3,020 | 1,386 | 656 | 108 | 8 |
| Person-years | 730,626 | 778,237 | 664,295 | 173,097 | 17,828 |
| Incidence (events/100000 person-years) | 413 | 178 | 99 | 62 | 45 |
| Adjusted HR (95% CI) | Ref. | 1.01 (1.00-1.02) | 1.01 (1.00-1.02) | 1.00 (0.99-1.02) | 0.99 (0.95-1.04) |
| Myocardial infarction |  |  |  |  |  |
| Events | 1,538 | 1,035 | 702 | 128 | 11 |
| Person-years | 764,629 | 792,660 | 671,027 | 174,312 | 17,876 |
| Incidence (events/100000 person-years) | 201 | 131 | 105 | 73 | 62 |
| Adjusted HR (95% CI) | Ref. | 1.01 (1.00-1.01) | 1.00 (0.99-1.02) | 1.00 (0.99-1.02) | 1.00 (0.96-1.05) |
| Stroke |  |  |  |  |  |
| Events | 8,696 | 6,064 | 3,901 | 732 | 54 |
| Person-years | 719,643 | 761,567 | 651,611 | 170,748 | 17,644 |
| Incidence (events/100000 person-years) | 1,208 | 796 | 599 | 429 | 306 |
| Adjusted HR (95% CI) | Ref. | 1.01 (1.00-1.02) | 1.02 (1.01-1.03) | 1.02 (1.00-1.03) | 1.01 (0.96-1.05) |
| Heart failure |  |  |  |  |  |
| Events | 4,136 | 2,632 | 1,708 | 311 | 18 |
| Person-years | 750,332 | 784,340 | 665,864 | 173,410 | 17,871 |
| Incidence (events/100000 person-years) | 551 | 336 | 257 | 179 | 101 |
| Adjusted HR (95% CI) | Ref. | 1.01 (1.00-1.02) | 1.01 (1.00-1.02) | 1.00 (0.99-1.02) | 1.00 (0.96-1.05) |

MACE, major adverse cardiovascular event; HR, hazard ratio, CI, confidence interval; Ref., reference.
